# Bacteria Exposed to Silver Nanoparticles Synthesized by Laser Ablation in Water: Modelling *E. coli* Growth and Inactivation

**DOI:** 10.3390/ma13030653

**Published:** 2020-02-01

**Authors:** Lucija Krce, Matilda Šprung, Ana Maravić, Polona Umek, Krešimir Salamon, Nikša Krstulović, Ivica Aviani

**Affiliations:** 1 Faculty of Science, Department of Physics, University of Split, Ruđera Boškovića 33, 21000 Split, Croatia; iaviani@pmfst.hr; 2 Faculty of Science, Department of Chemistry, University of Split, Ruđera Boškovića 33, 21000 Split, Croatia; msprung@pmfst.hr; 3 Faculty of Science, Department of Biology, University of Split, Ruđera Boškovića 33, 21000 Split, Croatia; amaravic@pmfst.hr; 4 Jožef Stefan Institute, Jamova cesta 39, 1000 Ljubljana, Slovenia; polona.umek@ijs.si; 5 Ruđer Bošković Institute, Bijenička cesta 54, 10 000 Zagreb, Croatia; Kresimir.Salamon@irb.hr; 6 Institute of Physics, Bijenička cesta 46, 10000 Zagreb, Croatia; niksak@ifs.hr

**Keywords:** laser synthesis of nanoparticles, silver nanoparticles, antibacterial activity, modeling bacterial growth

## Abstract

This study is aimed to better understand the bactericidal mode of action of silver nanoparticles. Here we present the production and characterization of laser-synthesized silver nanoparticles along with growth curves of bacteria treated at sub-minimal and minimal inhibitory concentrations, obtained by optical density measurements. The main effect of the treatment is the increase of the bacterial apparent lag time, which is very well described by the novel growth model as well as the entire growth curves for different concentrations. The main assumption of the model is that the treated bacteria uptake the nanoparticles and inactivate, which results in the decrease of both the nanoparticles and the bacteria concentrations. The lag assumes infinitive value for the minimal inhibitory concentration treatment. This apparent lag phase is not postponed bacterial growth. It is a dynamic state in which the bacterial growth and death rates are close in value. Our results strongly suggest that the predominant mode of antibacterial action of silver nanoparticles is the penetration inside the membrane.

## 1. Introduction

Antimicrobial resistance, being a global health problem [1], has been and currently is, an excellent motivator in the research of the antimicrobial activity of silver compounds, especially silver nanoparticles (AgNPs) [2,3,4,5,6]. The high surface-area-to-volume ratio of nanoparticles (NPs) provides different physical and chemical characteristics of NPs in comparison to bulk material or larger particles [7]. Many metallic and metallic oxide NPs show bactericidal potential towards different bacterial strains [7], but silver nanoparticles attract the most attention partially due to the antimicrobial activity of Ag^+^ ions [8]. Silver nanoparticles have been proven to be an excellent antibacterial agent against Gram-negative bacteria and a mild antibacterial agent against Gram-positive bacteria, possibly due to differences in their membrane structure [4]. They are widely available for purchase or are chemically produced [9,10,11]. Their bactericidal effect depends on their size [3], concentration [9], shape [2] and surface properties [8]. It has also been shown that AgNPs enhance the antibacterial potential of antibiotics compared to the drugs alone [12,13]. Reports of the impact of AgNPs on human health are still not numerous and are inconclusive as stated in the review paper [14]. It has been show that toxicity also depends on the physicochemical properties of nanoparticles [15] so it might be difficult to compare between toxicity research of AgNPs with different shapes and sizes at different concentrations.

However, significantly fewer reports on the bactericidal effect of nanoparticles produced by laser ablation in liquid (LAL) exist [16,17,18,19]. This technique, known as a ‘green synthesis’ technique [20], although other types of synthesis are considered to be green [21], enables nanoparticle production without chemical byproducts and stabilizing molecules [22].

The mode of antibacterial action of AgNPs is still not resolved [23] though many mechanisms incorporate the release of silver ions while some report formation of pits in the cell wall due to the nanoparticles accumulation [24]. It was reported that the mode of action is size-dependent: the bactericidal effect of AgNPs smaller than 10 nm is due to the nanoparticles, but the predominant mechanism of the larger ones might be the release of silver ions [25].

A bacterial cell concentration, as a function of time, obtained in a closed habitat, yields a growth curve generally consisting of four distinctive phases: lag phase, exponential phase, stationary phase and mortality phase as depicted in [26]. The lag phase, the delay of the exponential growth arising due to the changed growth conditions, has been a center of attention of many primary growth models [27,28,29]. This phase also seems to be induced when bacteria are treated at different sub-minimal inhibitory concentration (MIC) values of antibacterial substances [30,31,32,33], including silver ions and silver nanoparticles [11,34,35,36]. Commonly used approaches for bacterial growth modeling are the empirical Gompertz [37] and the rate logistic (Verhulst) models [26]. Scarce attempts have been made in modeling the bacterial growth in the presence of silver nanoparticles [38,39]. Recently published work [38] introduced a modified Gompertz model for bacterial isothermal growth data obtained at various AgNPs concentrations but without any interpretation at the microscopic level.

A relatively rarely investigated antibacterial mode of action of LAL-synthesized silver colloid, which is considered to be free of chemical byproducts, has a potential to reveal its bactericidal action without irrelevant or uncontrollable parameters. This has been our main motivation for this research.

We have synthetized silver nanoparticles by laser ablation, treated *E. coli* (*Escherichia coli*) and subsequently tracked the bacterial cell number in time through the optical density (OD) of the batch culture. We have aimed to explain the obtained OD growth curves of *E. coli* treated with different concentrations of laser produced AgNPs through modeling. Our objective was not just to give a mathematical description of the curves, but also, according to the modeling approach at a microscopic level, to explain the bacterial growing and dying mechanisms. We extended our model for non-treated *E. coli* cells [40], and described the bacterial growth through the system of three differential equations. These equations give the time change of bacterial, nutrients and nanoparticles concentrations. The model enabled us to understand the obtained growth phases, particularly the apparent lag phase and to propose the mode of antibacterial action of LAL-synthesized AgNPs which is one of the main prerequisites for efficient clinical trials and drug development. According to our knowledge, this approach has not been exploited.

## 2. Materials and Methods

### 2.1. Silver Colloid Production

Colloidal AgNPs were synthesized by laser ablation of silver target (purity >99.99%, GoodFellow, Huntingdon, UK) immersed in deionized water. The experimental setup is shown schematically in [41]. The silver target was immersed in a 30 mL beaker containing 25 mL of deionized water, while the water thickness above the target surface was 2 cm. The thickness of the water layer was kept constant during the laser ablation in order to keep the laser ablation efficiency constant [42]. The target was irradiated by a Nd:YAG laser using fundamental wavelength of 1064 nm and the laser pulse energy delivered to the target was 100 mJ giving fluence of 10 J/cm^2^. The number of delivered pulses was 5000 with the repetition rate of 5 Hz. The laser pulse was focused on the target surface using a 10 cm lens. The focal plane position was corrected after each 1000 pulses as the index of refraction of the produced colloid changed with the time of processing. This procedure was repeated four times in order to obtain 100 mL of colloid.

### 2.2. Silver Colloid Characterization

To determine the nanoparticle size, concentration, stability, composition and structure, the following measurements were performed.

The total volume of all AgNPs in the colloid was obtained from the volume of the craters that were created on the target surface during the laser ablation process. In order to determine the crater’s volume, the craters were studied with an optical microscope (Leitz Aristomet, Leica, Wetzlar, Germany) in a reflective illumination mode.

The ultraviolet-visible (UV–VIS) spectrum of synthesized Ag colloidal solutions was recorded in the wavelength range from 190−1100 nm using a UV–VIS spectrophotometer (Lambda 25, Perkin Elmer, Waltham, Massachusetts, USA).

Particles’ sizes and zeta potentials were determined by dynamic (DLS) and electrophoretic light scattering, respectively, using a Zetasizer Ultra (Malvern Panalytical, Malvern, UK). To avoid overestimation arising from the scattering of larger particles, the average particle size was obtained as the value at peak maximum of the number size distribution. The reported results are average of 10 measurements. The zeta potential result is reported as an average value of three measurements. The data processing was done by a ZS Xplorer 1.20 (Malvern Panalytical, Malvern, UK).

In order to perform structural characterization, the produced colloid was applied to a silicon substrate and left to air dry. This procedure was repeated several times resulting in an Ag film. The crystalline structure of the Ag films was investigated using a D5000 diffractometer (Siemens, Munich, Germany) in parallel beam geometry with Cu Kα radiation, a point detector and a collimator in front of the detector. Grazing incidence X-ray diffraction (GIXRD) scans were acquired with the constant incidence angle α_i_ of 1°, guaranteeing that the information contained in the collected signal covers the entire film thickness.

The morphology of the sample was investigated with a transmission electron microscope (TEM) (Jeol 2100, 200 kV, Jeol, Tokio, Japan). For TEM analyses, a specimen was prepared by adding 0.5 mL of the colloid to 0.5 mL of methanol and ultra-sonicated for a few minutes. Then one drop of the obtained dispersion was deposited on a lacey carbon film supported by a copper grid and dried in air. TEM nanoparticle size distribution was obtained by measuring the diameter of over 850 nanoparticles.

The specimen preparation for the atomic force microscopy (AFM) (Multimode 3, Digital Instruments/Bruker, Billerica, Massachusetts, USA) started by applying 5 µL of the colloid on a freshly cleaved mica sheet. The specimen was then left to air dry for 30 min. Imaging was done in contact mode at ambient conditions with probes having nominal spring constants of 0.12 N/m and 0.06 N/m (DNPS-10, Bruker, Billerica, Massachusetts, USA). AFM height images were analyzed using Gwyddion, an open source software for AFM data analysis. Height of each nanoparticle was taken as its diameter. Height measurements of over 170 nanoparticles yielded the particles size distribution.

Zeta potential, DLS and UV–VIS spectrum measurements were done immediately after the LAL synthesis, while the GIXRD measurements and microscopy studies were performed a few days later.

### 2.3. Minimal Inhibitory and Bactericidal Concentrations

To determine the minimal inhibitory concentration and the minimal bactericidal concentration the following experimental method and materials were used. The in vitro antibacterial activity of the synthesized Ag colloid was tested against the Gram-negative *E. coli* DH5α (kindly provided by Janoš Terzić Lab, School of Medicine, University of Split, Croatia).

DH5α is commonly used laboratory *E. coli* strain to maintain and amplify small plasmid DNA [43]. More importantly, it is often used in fundamental research of a wide range of new potential antibacterials [44,45,46,47] and even for the production of silver nanoparticles upon transformation [48]. Additionally, it seemed reasonable to model growth curves of this strain since we have thoroughly investigated its growth parameters [40].

Antibacterial activity was evaluated using the microdilution method according to the Clinical and Laboratory Standards Institute guidelines [49] and performed in 96-well microtiter plates. Nutritionally impoverished Luria-Bertani (LB) broth (5.0 g of tryptone, 2.5 g of yeast extract, and 5.0 g of NaCl per 1 L of deionized sterile water) was used in all experiments. This medium will be further referred as the master medium. An overnight culture of *E. coli* DH5α was diluted in the fresh master medium and grown for 60 min. Aliquots of bacterial suspension (50 µL) corresponding to 10^6^ colony forming units per milliliter (CFU/mL) were added to 50 μL of serial dilutions of the synthesized Ag colloid to a final load of 5 × 10^5^ CFU/mL per well. The silver colloid was briefly ultra-sonicated before adding it to the bacterial culture. Plates were incubated at 37 °C for 18 h before visual inspection. The MIC was read as the lowest concentration of the substance, showing no turbidity in the wells. For the minimal bactericidal concentration (MBC) determination, aliquots were taken from the wells corresponding to MIC and 2 × MIC and then plated on Mueller-Hinton agar (Biolife, Monza, Italy). After the incubation for 18 h at 37 °C, the MBC value was recorded as the concentration which caused ~99.9% of mortality in the starting inoculum. 

### 2.4. Optical Density Measurements

The microdilution method showed that the MIC value corresponded to the AgNPs concentration obtained at the 50% colloid volume share of the plate well. The bacteria were subsequently treated with different AgNPs concentrations (colloid volume shares) that were close to the MIC value. Growth curves of the treated bacteria were obtained by OD595 (optical density at 595 nm) measurements in a microtiter plate reader (BioTek ELx808 and BioTek Synergy HTX, BioTek Instruments, Winooski, Vermont, USA). Bacterial culture was prepared for the treatment as was for the MIC experiments. The colloid was added to each plate well so that it constituted 3% to 63% of the sample volume, while the rest of the volume consisted of bacterial suspension in the master medium. Before adding it to the bacterial suspension, the colloid was briefly ultrasonically agitated. The inoculum was kept at 5 × 10^5^ CFU/mL for all wells. The plate was shaken constantly at 37 °C. The OD was measured every 15 min for 24 h. All OD measurements were performed in triplicates. In order to obtain the bacterial concentration, the OD data were calibrated by viable cell counting. We found that 1 absorbance unit (a.u.) corresponds to 3.64 × 10^9^ CFU/mL.

### 2.5. Statistical Analysis

All experimental OD data were obtained in triplicate, and the results were expressed as the mean values and the maximal relative standard deviation was obtained for all growth curves. To compare the experimental data with the theoretical model, we have calculated the theoretical OD values for each experimental OD time point. The linear correlation between the corresponding experimental and theoretical data was quantified by Pearson correlation coefficient which was calculated for each couple of curves. The statistical analysis was performed using Microsoft Excel (Microsoft Office 2016, Microsoft Corporation, Redmond, Washington, USA).

## 3. Experimental Results

### 3.1. Colloid Characterization

The volume of a crater created during ablation was determined from a crater’s semi-profile as described in detail in [50]. Using this procedure, an average crater’s volume was determined since the colloidal suspension was made by four ablation procedures. The calculated average volume was 5.2 ± 0.8 × 10^6^ µm^3^ giving the total ablated volume in 4 craters V_tot_ = 2.1 ± 0.3 × 10^7^ µm^3^, which corresponded to the total volume of synthesized AgNPs. The ablated mass was dispersed in 100 mL of deionized water giving the mass concentration of 220 ± 32 µg/mL.

**Figure 1:**
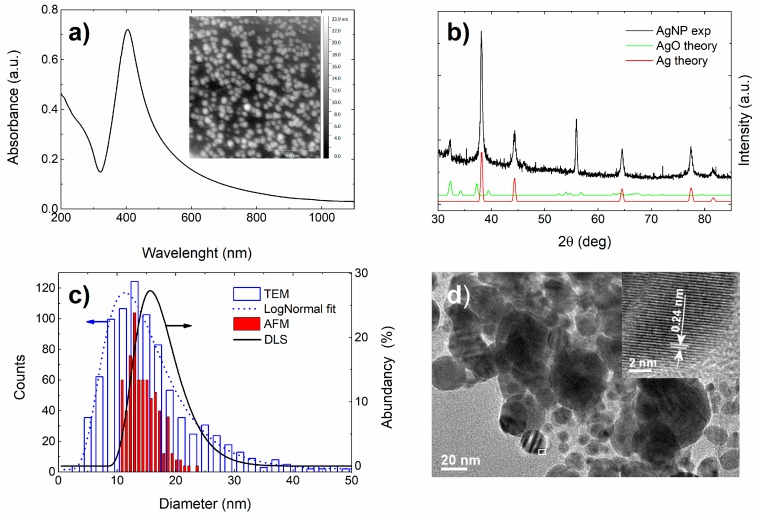
(**a**) UV–VIS photoabsorption spectrum of AgNPs exhibits a prominent characteristic peak at about 400 nm. The inset shows an AFM height image of a dried-out colloid sample reveling spherical shape of AgNPs, (**b**) GIXRD pattern obtained from AgNPs. Simulated patterns for the fcc-Ag and AgO phases are shown at the bottom. The peak at 2*θ* = 56° originates from the monocrystalline silicon substrate, (**c**) Size-distribution of AgNPs obtained from AFM images (red full column bar) with counts multiplied by a factor of 4 for better visibility and from TEM images (blue column bar) with the corresponding log-normal fit (blue dotted curve). The black full curve represents the size distribution obtained from the DLS measurements, (**d**) TEM image of AgNPs. The high-resolution TEM inset indicates the crystalline nature of AgNPs. The inset was taken in the area marked by a white frame.

Figure 1a shows an average UV–VIS spectrum for the four samples of silver colloids. The spectrum exhibits a sharp absorption peak in the visible range due to nanoparticle’s surface plasmon band [51]. The obtained maximum at 404 nm is typical for silver spherical stable colloidal nanoparticles in the diameter range of about 10 nm [52]. The zeta potential of nanoparticles is, as generally accepted, a measure of its stability [19], and for suspensions that are stabilized by electrostatic repulsion is required to be ± 30 mV at least [53]. Zeta potential of the produced silver colloidal solution was −(53.1 ± 1.1) mV. 

Figure 1b shows the GIXRD pattern for the Ag film together with the simulations for the fcc-Ag and the silver-oxide phases. The measured pattern matches with the fcc-Ag phase (ICDD card no. 00-004-0783). The appearance of all major Bragg peaks of Ag phase evidences randomly-oriented Ag crystallites. A relatively weak Bragg peak at 2*θ* = 32.18° proves that only a very small fraction of AgNP was oxidized.

The results of TEM analysis, presented in Figure 1d, support AFM (inset in Figure 1a) findings showing that the AgNPs are spherical-like. As seen in Figure 1c, their diameters were found mostly in the range from 3 nm to 40 nm (very rarely nanoparticles of up to 250 nm can be found). Nanoparticles are crystalline (inset to Figure 1), measured d-spacing of 0.24 nm corresponds to the (111) planes of Ag (ICDD card no. 00-004-0783) what is in the line with GIXRD findings. The AFM size distribution (Figure 1c) is obtained by measuring the height of the AgNPs (equal to the nanoparticle’s diameter, z-axis) since the lateral dimensions are not to be taken into account due to the AFM tip dilation [54]. An average AgNP diameter obtained from the AFM images is 13.1 nm, while the TEM images revealed the diameter to be 11.3 nm (the discrepancy is possibly due to better resolution of the TEM microscope).

DLS measurements reveal that the dynamic AgNP diameter is 15.6 nm (black line given in Figure 1c). However, all size distributions are relatively broad ranging from about 3 nm to 50 nm with the maximum at around 10−15 nm. The TEM size-distribution follows a log-normal (Log*N*) distribution fit represented by the blue dotted line. Log*N* fit is often used to describe the size-distribution of NPs synthesized from gaseous phase, which is the case in NP synthesis by LAL. It is applied whenever particle growth depends on the diffusion and drift of atoms to a growth zone of the nanoparticles [55], while the final distribution is determined with the available growth time of the nanoparticles [56]. The average volume of a single nanoparticle can be determined from the average diameter of the NPs. Dividing the ablated volume of silver, dispersed in 100 mL of water, by the average nanoparticle volume, one obtains the concentration of AgNPs to be 1.2 ± 0.2 × 10^11^ mL^−1^ as obtained from the TEM diameter. The procedure of how to determine the nanoparticle concentration is described in detail in [57]. 

### 3.2. Bacteria Treatment Results

**Figure 2:**
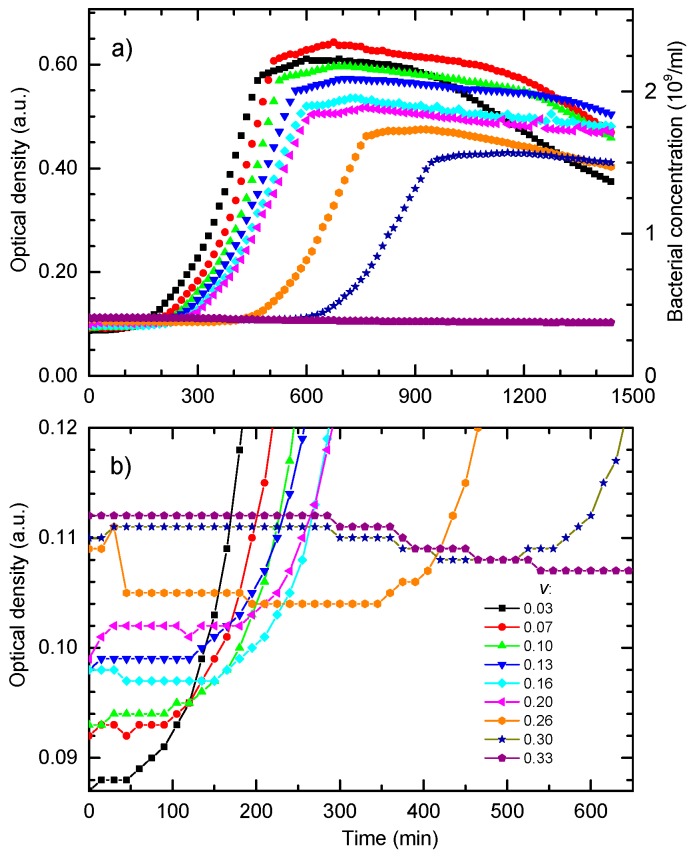
(**a**) Growth data of *E. coli* treated with different concentrations of silver nanoparticles, i.e., different colloid volume shares *v*. The OD in absorbance units is given on the left axis and the corresponding bacterial concentrations in CFU/mL are given on the right axes. (**b**) The first 650 min of the experimental OD data given in Figure 2a.

The MIC and MBC values obtained by the microdilution assay for the produced Ag colloid against *E. coli* DH5α were resolved to be (110 ± 16) µg/mL. To study how a near-MIC treatment influences bacterial growth, the OD growth experiments were made. The raw OD bacterial growth data at 595 nm for the eight sub-MIC and MIC treatments are given in Figure 2a, where the OD signal is proportional to the concentration of bacteria. The maximal relative standard deviation for the three replicates was 4.5% of the OD signal for all growth curves. The numbers given in the legend are the volume shares *v* of the colloid in the batch culture, and are proportional to the concentration of the NPs. The concentration of the NPs for each treatment is obtained by multiplying the concentration of NPs in the produced colloid by *v*. As seen in Figure 2a, the main effects of increasing the AgNPs concentration are: increase of the baseline, increase of the bacterial lag time and the reduction of the maximal OD. 

The growth curve for *v* = 0.30 exhibits the lowest maximal OD and the largest lag time. For *v* ≥ 0.33 bacterial growth is completely suppressed, that is when the NPs concentration in the batch exceeds the value of 73 ± 11 µg/mL. This value is close to the MIC/MBC obtained by microdilution. Note that, for the microdilution assay, a serial dilution of the produced colloid was made, thus reducing the AgNPs concentration in the successive wells by the factor of 2. In comparison, the OD data gave finer and more accurate results. 

The details of the first 650 min of growth are shown in Figure 2b. They reveal the bacterial population dynamics during the lag phase. Growth curves obtained for bacteria treated with higher concentrations of NPs show an increase of the initial optical density and appearance of the minimum in the OD before the growth of bacteria prevails. The bacterial inactivation curve obtained for *v* = 0.33 exhibits a constant decrease in the OD. 

The OD data given in Figure 2a,b have motivated us to develop a model of bacterial growth in the presence of the antimicrobial agent.

## 4. Model Evolution

**Figure 3:**
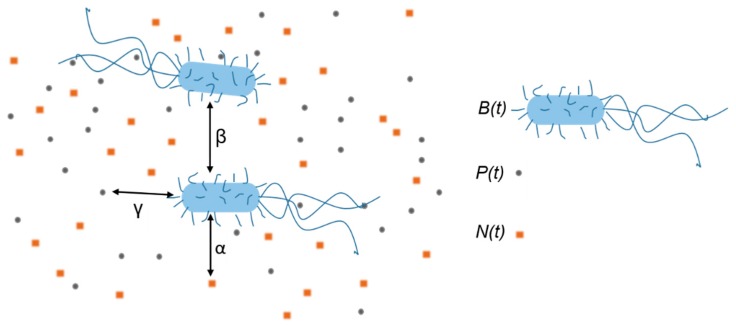
The schematic representation of the batch system as described by our model. The squares and circles represent the nutrients and nanoparticles “packages” that correspond to a single bacterium creation or inactivation, respectively. Interactions are represented by double-head arrows and marked by their interaction strengths *α*, *β*, and *γ*. They describe the following occurrences: Bacteria (i) consume the nutrients, (ii) interact with other bacteria, and (iii) they uptake nanoparticles.

The model we apply here is an extension of the interaction-based model that successfully describes growth and inactivation of untreated *E. coli* cells [40]. The sketch of the model’s mechanism, i.e., included interactions within the system when bacteria are treated with the AgNPs, is given in Figure 3. Bacteria consume nutrients and divide which leads to the increase of the bacterial concentration *B*(*t*) and the decrease of the nutrients concentration *N*(*t*). Bacteria also interact with the nanoparticles which leads to the decrease of the AgNPs concentration *P*(*t*) and the bacterial concentration *B*(*t*). We find convenient to express both *N*(*t*) and *P*(*t*) in bacteria-equivalent units, that is in “packages” per bacterium as described below.

If *m*_N_/*V* is the mass concentration of nutrients, where *m*_N_ is the mass of nutrients and *V* the volume of the sample, and *m*_N1_ is the quantity of nutrients needed for a single bacterium growth, then *N*(*t*) = (*m*_N_/*m*_N1_)/*V* is concentration of the bacteria that could be produced with the given nutrients concentration. Similarly, if *N*_p_/*V* is the concentration of AgNPs, where *N*_p_ is the number of the AgNPs in the sample and *N*_p1_ is their number needed for a single bacterium inactivation, then *P*(*t*) = (*N*_p_/*N*_p1_)/*V* is the concentration of the bacteria that could be inactivated with the given concentration of AgNPs.

The bacterium-nutrient interaction strength *α* and bacterium-nanoparticle interaction strength *γ* describe the positive influence of the nutrients and the negative influence of the AgNPs on the bacterial population, respectively. The bacterium-bacterium interaction strength *β* describes the negative bacterial influence on the bacterial growth such as: the presence of metabolite waste, bacterial competition for nutrients, and possibly lack of space. The model is mathematically expressed by the system of three mutually dependent non-linear differential equations:

(1) $$\frac{d}{dt}B\left(t\right)=\alpha \cdot B\left(t\right)\cdot N\left(t\right)-\beta \cdot B{\left(t\right)}^{2}-\gamma \cdot B\left(t\right)\cdot P\left(t\right)$$

(2) $$\frac{d}{dt}N\left(t\right)=-\alpha \cdot B\left(t\right)\cdot N\left(t\right)$$

(3) $$\frac{d}{dt}P\left(t\right)=-\gamma \cdot B\left(t\right)\cdot P\left(t\right).$$

The first equation describes the time change of the bacterial concentration. The bacterial concentration increases due to the presence of nutrients, and decreases due to the presence of other bacteria and AgNPs. The second and third equations describe the decrease of nutrients and AgNPs concentrations in time, respectively, with the presumption that their concentration is changed only because of the bacterial consumption. If the concentration of nutrients is changed only because of the bacterial consumption, then the number of bacteria produced in unit of time should reduce the number of the nutrients packages for the same amount. This assumption is taken into account by putting the same coefficient *α* in Equations (1) and (2). The corresponding rates of change are equal in size but opposite in sign. Similarly, if the colloid is stable, the concentration of AgNPs is changed only because of bacterial uptake. The list of the parameters included in the model is as follows:

*t*—time
*B*(*t*)—bacterial concentration
*B*_0_—inoculum size
*N*(*t*)—nutrients concentration
*N*_M_—master growth medium nutrients concentration
*P*(*t*)—AgNPs concentration
*P*_M_—produced colloid AgNPs concentration
*x*—initial nutrients dilution factor
*v*—initial volume shares of the colloid
*α*(*x*)—bacterium–nutrients interaction strength parameter
*β*(*x*)—bacterium–bacterium interaction strength parameter
*γ*—bacterium-nanoparticle interaction strength

The number of bacteria inactivated in a unit of time, due to the AgNPs, should reduce the number of the AgNPs packages for the same amount, which is taken into account by putting the same coefficient *γ* in Equations (1) and (3). The bacterial growth is determined by the initial bacterial concentration (inoculum) *B*(0), initial concentration of nutrients “packages” *N*(0) and initial nanoparticles “packages” concentration *P*(0). In our experiments, the inoculum was kept constant, while the AgNPs concentration was changed by mixing the appropriate volume *V*_P_ of the colloid, that has the AgNPs concentration *P*_M_, with the volume of the appropriately inoculated master growth medium *V*_N_, that has nutrients concentration *N*_M_, so that the total volume of the batch culture *V* = *V*_N_ + *V*_P_ is kept constant. In this way, the initial volume shares of the colloid *v* =*V*_P_/*V* = *P*(0)/*P*_M_ and of the growth medium *x* = *V*_N_/*V* = *N*(0)/*N*_M_ are varied so that the condition:

(4) x+v=1

is fulfilled. Therefore, the initial conditions are defined by Equations (5)–(7):

(5) $$B\left(0\right)\equiv B_{0}$$

(6) $$P\left(0\right)=P_{M}\cdot v$$

(7) $$N\left(0\right)=N_{M}\cdot x=N_{M}\cdot \left(1-v\right).$$

In our previous work [40], we found that the interaction strengths *α* and *β* depend on the initial nutrients concentration with the dependence in the form of a rational function *const.*/(0.093 + *x*). For our system, the best fits are obtained for *α* = *α* (*x*) = (0.0295 min^−1^∙a.u.)/(0.093 + *x*) and *β* = *β* (*x*) = (0.00045 min^−1^∙a.u.)/(0.093 + *x*). According to Equation (4), *α* and *β* can also be expressed in terms of the initial colloid volume share *v* giving *α* (*v*) = (0.0295 min^−1^∙a.u.)/(0.093 + 1 − *v*) and *β* (*v*) = (0.00045 min^−1^∙a.u.)/(0.093 + 1 − *v*).

**Figure 4:**
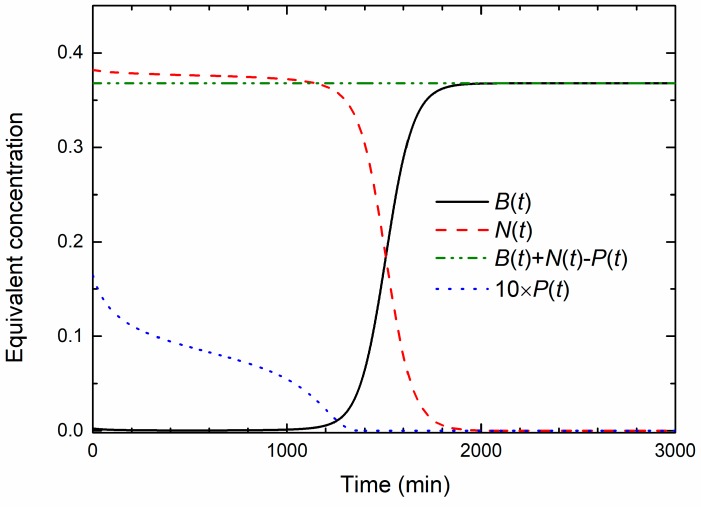
Time change of bacterial concentration *B*(*t*) and the equivalent concentrations of nutrients *N*(*t*) and nanoparticles *P*(*t*) as obtained from the solution of Equations (1)–(3). Note the conservation of the total growth capacity *B*(*t*) + *N*(*t*) − *P*(*t*) = *const*.

Solutions of Equations (1)–(3), for *B*_0_ = 0.0025 a.u. *N*_M_ = 0.57 a.u., *P*_M_ = 0.05 a.u., *v* = 0.33, *γ* = 1.65 and *β* = 0 are plotted in Figure 4. As will be shown latter, these values correspond to the experimentally observed OD data obtained for the MIC treated culture. Note that all concentrations are expressed in terms of a.u., so that calculated values can be compared to the experimental data. In order to make the NPs’ concentration change visibly on the given scale, the *P*(*t*) data were multiplied by a factor of 10. Additionally, to emphasize the main results of the model, parameter *β*, which is responsible for the population decay in the death phase, is set to zero. At the beginning, the bacterial concentration slightly decreases, then acquires a broad minimum and then slowly increases, so that the bacterial population remains below the inoculum size up to 1000 min approximately. In that period, both the nutrients and the NPs’ concentrations decrease, exhibiting similar functional dependence. Obviously, a dynamic equilibrium exists in which approximately an equal number of bacteria divide, because of the nutrients consumption, and die due to the uptake of the nanoparticles. The exponential bacterial growth appears only if the concentration of nanoparticles falls below some critical value. As a result of that process, the exponential growth phase is significantly postponed. For *t* > 1000 min, the onset of the exponential growth phase is seen and the stationary phase is reached for *t* > 2000 min. 

As seen from Figure 4, the sum *B*(*t*) + *N*(*t*) − *P*(*t*) = *const.* holds at all times during the growth. This sum is also the final concentration of the bacterial population; thus it represents the total capacity of the system. 

**Figure 5:**
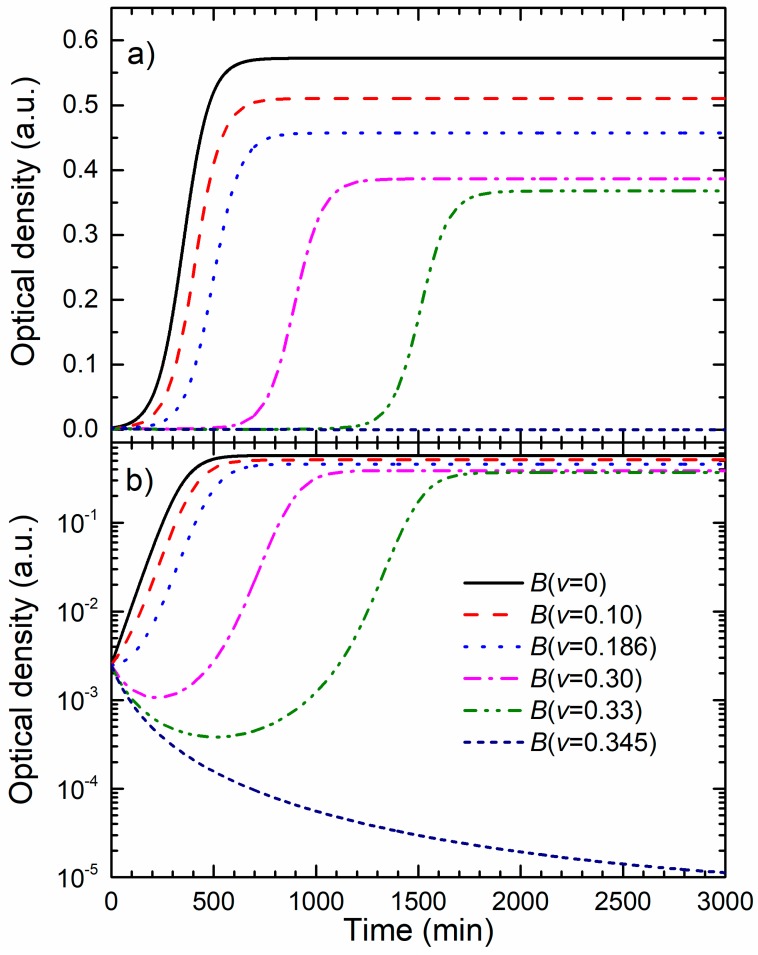
(**a**) Bacterial population obtained from solutions of Equations (1)–(3) for *β* = 0 for different colloid volume shares *v*. The increase of the colloid volume share reduces the maximal bacterial concentration and postpones the exponential bacterial growth. (**b**) The same data given in logarithmic scale, exhibiting the population dynamic of the lag phase.

To further elucidate the proposed model, it is useful to inspect the bacterial growth behavior upon treatment with different initial NPs concentrations. Figure 5a shows bacterial growth curves calculated for different NPs concentrations while all other parameters were the same as for the data given in Figure 4. Bacterial growth for non-treated cells is represented by the full curve. The growth curves for several sub-MIC concentrations show that the main effect of the NPs concentration increase is the delay of the exponential growth phase. For volume share *v* = 0.345 given in dotted curve, bacteria do not go into the exponential phase. This behavior is in accordance with the experimental data given in Figure 2.

The decrease of the maximal population size with the increase of the colloid volume share is mostly related to the dilution of the master growth medium and the decrease of the initial nutrients concentration, i.e., the total growth capacity. 

Figure 5b shows the growth data from Figure 5a presented in the logarithmic OD scale. In this representation, it can be found that the postponed exponential bacterial growth is not a classic lag in which bacteria do not divide. This state can be characterized as a dynamic lag phase that is close to the steady state because the bacterial growth rate is close to the bacterial death rate. As expected, cells in the non-treated sample grow exponentially from the beginning, and the exponential growth of the cells treated with the colloid volume shares of 0.10 and 0.186 is postponed.

For higher nanoparticle concentrations, *v* = 0.30 and *v* = 0.33, the bacterial population firstly decreases, and develops a broad minimum, before the exponential growth prevails. The minimum is related to the prevailed onset of the exponential growth. To determine the lethal nanoparticle dosage, the concentration range in which a minimum of *B*(*t*) develops and disappears should be found analytically. We found that the minimum disappears for *v* ≥ 0.345 treatments for which the bacterial population decreases continually. This value is in excellent agreement with the experimentally-obtained MBC concentration.

According to the model, the decrease of bacterial concentration is due to the uptake of the AgNPs, which causes bacterial death. In this process, the nanoparticles are removed from the habitat. This is in line with the study done by Sondi and Salopek Sondi [8] where TEM analysis clearly showed that the nanoparticles were accumulated in the membrane, while some of them successfully penetrated into the cells. When the concentration of the NPs falls below a certain level, the rest of the viable bacteria start to divide. 

## 5. Data Analysis and Discussion

There is a wide range of reported MICs of silver nanoparticles, typically between 1 and 100 µg/mL [3,9,39,58,59]. Since the bactericidal effect depends on nanoparticles’ physical and chemical properties, the bacterial strain and growth conditions, it is reasonable to conclude that the obtained MIC value of 73 ± 11 µg/mL falls within the reported range. 

To compare the model with the experimental data from Figure 2a,b, the contributions of nutrients, nanoparticles, and cells to the measured OD data should be taken into account. We neglect the contribution of nutrients in accord to our previous findings [40], where the change in nutrients concentration did not provide observable change in the initial OD signal for the nutrients concentrations described here. The total OD signal can then be written as the sum of the constant contribution of the well plate and the medium *c*_m_ and the time and volume-share dependent contributions of bacterial *c*_b_*B*(*t*,*v*) and nanoparticles *c*_p_*P*(*t*,*v*) concentrations:

(8) $$OD\left(t,v\right)=c_{m}+c_{b}\cdot B\left(t,v\right)+c_{p}\cdot P\left(t,v\right)$$

The contribution of AgNPs is in accordance with Figure 1a that shows absorbance of the silver nanoparticles at 595 nm, the wavelength used in our OD experiments.

The constants *c*_b_ and *c*_p_ reflect the light scattering/absorbance intensity for the bacteria and for the nanoparticles, respectively. To compare the experimental and theoretical growth curves, these constants are determined. The constants are obtained from the two limiting cases: the beginning of the growth, where *B*(0, *v*) ≈ 0, and from the maximum of the population, where *P*(*t*, *v*) ≈ 0.

**Figure 6:**
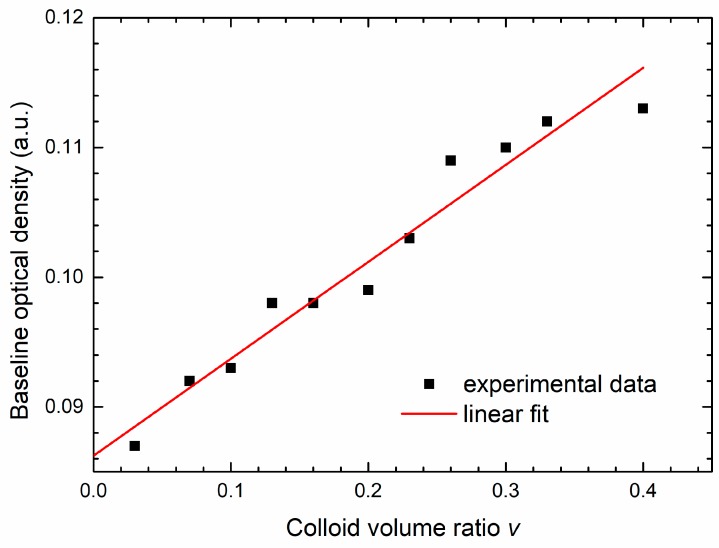
Initial OD (baseline) of the experimental growth curves given in Figure 2a, as a function of the colloid volume share v, is given by squares. Linear fit obtained for these data, given by the straight line, is the contribution of the silver nanoparticles to the total OD.

The contribution of the NPs to the total OD signal is observed at the beginning of the growth, while the bacterial concentration is very low and not visible in the OD signal, so the condition *B*(0,*v*) = 0 in Equation (8) is fulfilled and we assume that the whole change in the OD signal is due to the change of the NPs concentration. The contribution of the AgNPs to the OD signal is obtained from the concentration dependence of the initial OD(0, *v*) obtained for different growth curves. These data are shown in Figure 6. The line is the linear fit to the data given by the expression:

(9) OD(0,v)=(0.086+0.074·v) a.u.

where the intercept *c*_m_ = 0.086 a.u. is the absorbance of the well plate and the medium. The coefficient *c*_p_ = 0.074 a.u. is obtained from the slope of the linear fit. We see that maximal overall contribution of AgNPs to the total OD is of the order of 0.02 a.u. and is much less than the maximum bacteria OD, which is around 0.5 a.u.

Since NPs are taken up by the bacterial cells, the concentration of the nanoparticles decreases so from the exponential phase and onwards it becomes negligible, in comparison to the bacterial concentration, so we can put *P* (*t*, *v*) = 0. Equation (8) becomes:

(10) $$OD\left(t,v\right)=c_{m}+c_{b}\cdot B\left(t,v\right)$$

and we can compare the OD data with the calculated bacterial concentration.

Equation (10) shows that if we subtract *c*_m_ from the experimental *OD* (*t*, *v*) data and set *c*_b_ to 1, there is one-to-one correspondence between the theoretical calculations and experimental data. In other words, the calculated, theoretical data, are expressed in a.u. Note that the theoretical concentrations of nutrients and nanoparticles are calculated in terms of bacterial equivalents, as explained before, and are also given in a.u.

**Figure 7:**
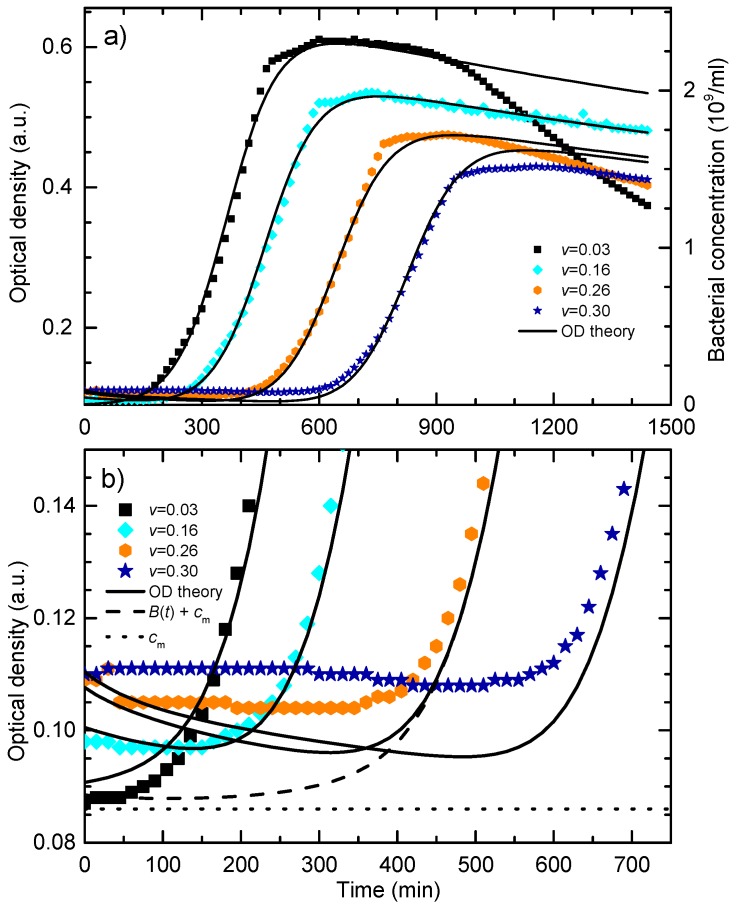
(**a**) Comparison of the experimental curves (symbols) with theoretical fits for different *v* values (lines). (**b**) the first 700 min of the OD signal from Figure 7a. The symbols are the raw experimental data for the different colloid volume shares and the full lines are the theoretical fits as given by Equations (1)–(3) and Equation (12). The dashed line is the theoretical bacterial concentration *B*(*t*) for *v* = 0.26 summed with the constant *c*_m_ = 0.086. The dotted line is *c*_m_ = 0.086 a.u., i.e., the optical density of the well plate and the master medium.

When modelling the experimental curves in Figure 7a, we found that the best fits are obtained for the initial nanoparticles concentration *P*(0, *v*) = 0.05 × *v* a.u.. Substituting this expression for *v* in Equation (9) gives:

(11) $$OD_{th}\left(0,v\right)=0.086\;a.u.+1.5\cdot P\left(0,v\right).$$

Equation (11) gives calculated initial OD for different growth curves as a function of the initial colloid volume share *v*, i.e., concentration of nanoparticles. It reveals that one “package” of the AgNPs needed to annihilate a single bacterium contributes 1.5 times more to the total OD when compared to the OD contribution of a single bacterium. This dependence is used to describe time evolution of the OD for a growth curve for which the nanoparticles concentration is time-dependent.

As a result, Equation (8) assumes the form:

(12) $$OD_{th}\left(t\right)=0.086\;a.u.+B\left(t\right)+1.5\cdot P\left(t\right).$$

This allows us to model the obtained experimental OD data with more accuracy. To compare the experimental and theoretical data, the parameters were adjusted and the solutions of Equations (1)–(3) were calculated in bacteria-concentration equivalent absorbance units. In accordance with the Equation (12) the calculated nanoparticles concentration is multiplied by the factor of 1.5. The comparison of the experimental and theoretical OD response of bacteria and nanoparticles for the four representative experimental growth curves from Figure 2a is given in Figure 7a. The symbols are the experimental data while the lines are the theoretical fits according to the solutions of the Equations (1)–(3) and OD contributions given by Equation (12).

The fitting procedure was the following. Firstly, the growth curve for the colloid volume share *v* = 0.16 was fitted in a way that only parameter *v* = 0.16 was fixed and all other parameters in the system of Equations (1)–(3) were varied. This curve was chosen merely because it is in the middle of the treatment range. Initially, to obtain *P*_M_, the contribution of nanoparticles in the OD signal was neglected, but for further iterations Equation (12) was used to compare the fit with the experimental data. The least-square fit was obtained for the following parameters set: *B*_0_ = 0.0025, *P*_M_ = 0.05, *N_M_*= 0.57, *γ* = 1.65. When fitting the other curves, only the parameter *v* was changed while all other model parameters were kept at the same values as for the *v* = 0.16 sample.

It can be seen that the theoretical curves fit the experimental data for the lag and the exponential phase very well, i.e., the model completely describes the nanoparticles concentration-dependent postponed bacterial growth. The obtained correlation coefficients for the corresponding sets of data are: 0.96 for *v* = 0.03 curves, 0.99 for *v* = 0.16 curves, 0.99 for *v* = 0.26 curves, and 0.99 for *v* = 0.30 curves.

The postponed bacterial growth for cultures treated with AgNPs is found in many published data [9,10,38,39,60]. Our model, however, less successfully describes the reduction of the maximal OD for higher nanoparticles concentration, as well as the time-dependent decrease of the bacterial population in the death phase. Lowering of the maximal OD is reported in many studies [9,10,11,38] while some report relatively unaffected max OD value for treatments with spherical silver nanoparticles [39,61]. In our case, the decrease of the maximal OD is mostly related to the experimental design. Notably, the maximal bacterial population depends on the initial concentration of nutrients [40]. In the experiment, the NPs concentration is changed by replacing the growth medium with the produced colloid. Consequently, the samples with the higher AgNPs concentration had lower initial concentration of nutrients and thus developed lower maximal number of bacterial cells.

It is also noticeable that for higher AgNP concentrations, the experimental maximal OD is systematically lower than the calculated value. The drop down of the maximal OD could be due to the return of AgNPs back to the habitat, caused by the decomposition of the dead cells. In other words, it is reasonable to presume that a dead bacterium decomposes and releases the nanoparticles back into the batch so that some nanoparticles might be used more than once. However, these considerations demand further investigation and modeling of the system.

The data also show that the AgNPs treatment does not change the bacterial growth rate. A weak growth rate reduction can be attributed to the change in nutrients concentration as we found for untreated cells [40]. Published data show a discrepancy regarding this issue: some report relatively unaffected growth rates [39,60] while others report growth rate reductions [11,38]. To resume, we found that the change in the observed maximum OD and the growth rate is due to the change in the initial nutrients concentration and not due to the influence of the AgNPs.

Figure 7b presents details of the data from Figure 7a at the beginning of the growth. The symbols are the raw experimental data and the full lines are the theoretical fits for the different colloid volume shares, as given by Equations (1)−(3) and Equation (12). At this scale, all the contributions to the optical density signal can be resolved. This is demonstrated for the sample with the colloid volume share *v* = 0.26. The dotted line is the constant *c*_m_ = 0.086 a.u., i.e., the OD of the well plate and the master medium. The difference between the dashed and dotted line is the theoretical bacterial contribution *B*(*t*) and the difference between the dashed and the full line is the theoretical contribution of the nanoparticles *P*(*t*). As can be seen in the figure, for *t* > 500 min this difference is practically zero because *P*(*t*) falls to zero. When *B*(*t*) and *P*(*t*) curves are compared, it is noticeable that the prominent decrease in the OD value, before the bacterial concentration OD signal prevails, can only be attributed to the reduction of the nanoparticle concentration in the habitat due to the bacterial uptake. From Figure 7b, we also see to what extent the theoretical model can describe the experimental data. Generally, it can be noted that the agreement with the experiment is better for lower nanoparticles concentrations, *v* = 0.03 and *v* = 0.16, then for the higher ones. For higher nanoparticle concentrations, theoretical curves exhibit a more pronounced minimum in the OD when compared to the experiment. This could be because the nanoparticles that were adhered to the bacterial surface still absorb some of the light and thus still contribute to the measured OD signal. However, the theoretical curves follow the trend of the OD decrease before bacterial growth prevails which is observed in the experimental data.

**Figure 8:**
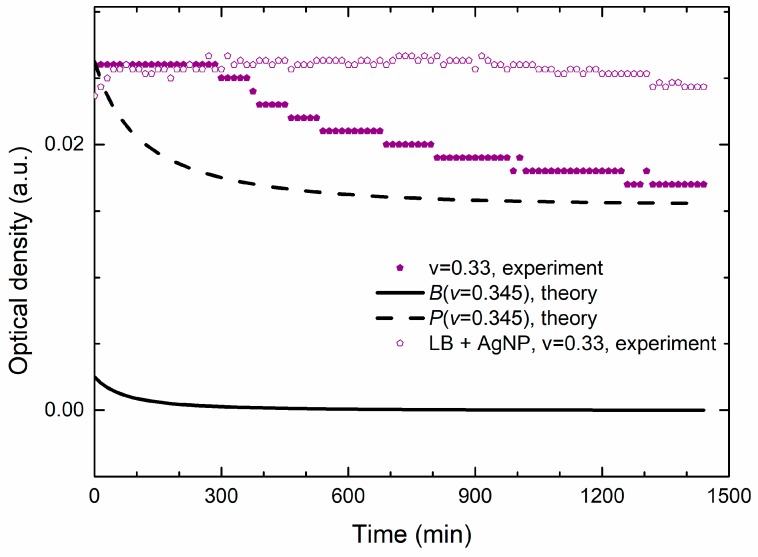
Growth curves of bacteria treated with the MBC concentration. The full symbols are the experimental OD data for bacterial *v* = 0.33 treatment (*c*_m_ value was subtracted), i.e., the AgNPs’ OD signal. The full and the dashed lines are the theoretical data for bacterial and for nanoparticle concentrations, respectively, for *v* = 0.345. All the curves exhibit a continuous drop of OD for the given time frame. The open symbols are the experimental data obtained for *v* = 0.33 mixture of LB growth medium and AgNPs.

The theoretical calculations show that, at the beginning of the growth, the nanoparticles contribution to the OD dominates over the bacterial and that the decrease of the total OD, as seen in Figure 7b and Figure 8, comes from the decrease of the nanoparticles concentration in the batch. It is reasonable to presume that this decrease is due to the uptake of the nanoparticles by the bacteria because the nanoparticles that enter the bacteria are no longer visible to the spectrophotometer. Our results are supported by the TEM data which clearly showed that the AgNPs have penetrated the membrane/cell [9].

When modeling non-treated curves, we have shown [40] that the bacterial OD could not be observed at the initial time by the utilized spectrophotometer for the inoculum size used in these experiments. This indicates that the initial decrease of OD given in Figure 7b, cannot be attributed to the inactivation of the bacterial cells. When it comes to the MIC/MBC value, there is a small discrepancy between the experimental and theoretical data. From the experiment we found that the MIC/MBC value is at *v* = 0.33 and the theoretical model gives *v* = 0.345. 

Figure 8 shows the experimental and theoretical growth data for the MIC/MBC concentration. We see that the theoretical OD data are lower than the measured OD, but they describe well the time dependence of the measured OD. Additionally, Figure 8 reveals that the nanoparticles contribution to the OD signal is approximately an order of magnitude greater than the contribution of bacteria. The bacterial concentration also exhibits a decrease but on the scale that is comparable with the sensitivity of the spectrophotometer (about 0.001 a.u).

It is generally accepted that the silver ions (Ag^+^) are, to some extent, released by AgNPs and there are opinions that these Ag^+^ ions are mostly responsible for the antibacterial effect. According to the literature [62], silver ions are likely to interact with chloride ions (Cl^−^) present in the bacterial growth medium and can cause precipitation of silver ions as AgCl(s). Therefore, there is a possibility that the decrease in the OD signal found at the lethal concentration of AgNPs appears due to the AgNPs dissolution in the growth medium.

However, our experimental data obtained for the same non-inoculated sample, given by open symbols in Figure 8, exhibits almost constant OD which does not support the Ag^+^ release mechanism as the main cause of the obtained decrease of the OD, which is expected to appear even without the cells. Since the OD decrease does not appear if there are no bacteria in the batch, we conclude that these data support the hypothesis of our model, according to which the OD decreases because of the bacterial uptake of the nanoparticles.

This supports our statement that, for the lethal treatment, the OD signal measures mostly the change in the concentration of the nanoparticles due to the bacterial uptake.

It should be emphasized that the lag phase was not observed in our experiments with the non-treated bacteria, which were prepared according to the same protocol [40] so the “traditional” lag is not included in the model. Figure 7b clearly shows that the postponed exponential growth is not due to the classical lag, i.e., the absence of the bacterial division, and should be considered as a dynamic lag phase. According to the model, Figure 4 shows that the nutrients consumption continues even when the bacterial concentration is being reduced. Thus, during this dynamic lag phase, bacteria are dividing and being inactivated by the AgNPs at the same time. The duration of the dynamic lag phase depends on the nanoparticles concentration. We find that the time interval measured from the beginning of the growth to the inflection point (the point at which the growth is maximal) is the most reliable measure for comparing the experimental and theoretical data. This time is easily determined from the maximum of the time derivative of the growth curve. We propose that this time should be called the dynamic lag time. The dynamic lag phase includes the bacterial division and inactivation. 

**Figure 9:**
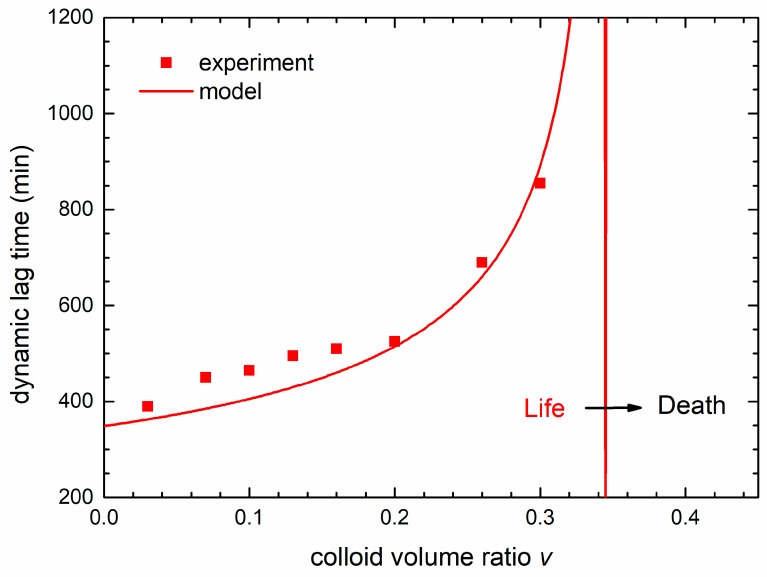
The dependence of the dynamic lag time on the colloid volume share *v*. The squares are the data obtained from the experimental growth curves and the solid line are the calculated data obtained from the theoretical model. The limit between bacterial life and death conditions (lethal treatment) is denoted by the vertical dashed line.

The symbols in Figure 9 are the dynamic lag times obtained from the maximum of the derivative of the experimental OD curves from Figure 2a for different colloid volume shares *v*. The full line, obtained from the derivative of the theoretical curves, follows the experimental data very well. We see that the dynamical lag time increases with the nanoparticles concentration and diverges (becomes infinite) at the lethal colloid volume share *v* = 0.345. The limit between bacterial life and death conditions (lethal treatment) is denoted by the vertical line. In fact, Figure 9 represents a transition between life and death conditions, transforming the bacterial habitat from the one that enables bacterial growth to a habitat in which the bacteria growth is suppressed. 

Since there are rare attempts on modeling the bacterial growth in the presence of nanomaterial in general, we find this model a good start for future developments. It is important to note that further validation of this model should be conducted for different bacterial strains growing under different conditions. The model might also be applicable if other antibacterial agents are utilized as long as they are detectable via spectrophotometer.

## 6. Conclusions

The main goal of this study is better understanding of bactericidal mode of action of silver nanoparticles. The silver nanoparticle colloid was synthetized by laser ablation in deionized water. To determine the NPs stability, composition, structure, size and concentration, UV–VIS, zeta potential, GIXRD, DLS, AFM, and TEM measurements were performed. The concentration of the AgNPs in the colloid was estimated from the volume of the ejected material. The colloid proved to be stable having spherical NPs with the average diameter of ~11.3 nm and the log-normal size distribution. The GIXRD results revealed the fcc-Ag crystallites and very weak presence of the silver oxide phase.

The colloid was tested against *E. coli* DH5α cells which showed sensitivity towards the produced colloid. The AgNPs MIC/MBC values were 73 ± 11 µg/mL. Growth curves of bacteria treated with sub-MIC and MIC AgNP concentrations were obtained via the OD measurements. 

The main effects of the treatment are the increase of the baseline of the growth curve, increase of the apparent lag time, and reduction of the maximal OD, without significant change of the growth rate. Growth curves of bacteria treated with higher sub-MIC concentrations exhibit a minimum, before the exponential growth prevails. For concentrations higher than the MIC, the growth is suppressed and the OD constantly decreases.

In order to analyze the data, we designed a novel growth model as an extension of the interaction-based model for the non-treated cells [40]. The main assumption of the model is that treated bacteria uptake the nanoparticles, which results in the decrease of the both the nanoparticles and the bacteria concentrations. The time change of the bacterial, nutrients, and nanoparticles concentrations depends on the strengths of the bacteria–nutrients, bacteria–bacteria, and bacteria–nanoparticles interactions.

We have shown that the experimental growth data reflect the concentration of both bacterial cells and nanoparticles. The contribution of the nanoparticles was clearly observed at the beginning of the growth, when the nanoparticles OD signal dominates the bacterial one. The signal was found to be constant for the non-inoculated sample and it decreased for the lethal bacterial treatment because AgNPs enter the bacteria. This also brings us to the conclusion that, for the lethal treatment, the decrease in OD mostly reflects the change in the nanoparticles concentration.

The model completely describes concentration-dependent postponed growth, with very good fits for the apparent lag and the exponential phase. It also describes the reduction of the maximal population with the colloid volume share increase and the decrease of the bacterial population in the death phase. According to the model, the postponed exponential growth is not due to a classic lag in which bacteria do not divide. This state can be characterized as a dynamic lag phase, or a near steady state for which the bacterial growth rate is close to the bacterial death rate. In the lag phase bacteria are dividing due to nutrients consumption, and being inactivated due to the uptake of the silver nanoparticles, at the same time. The duration of the dynamic lag time increases with the nanoparticles concentration and becomes infinite at the lethal concentration, representing a transition between bacterial life and death conditions.

To conclude, the main contributions of this research may be emphasized as follows. The observed lag phase induced by AgNPs is not postponed bacterial growth and the predominant mode of antibacterial action of AgNPs is their penetration inside the cell.
